# Study on the Variation of Resistance Factor of Polymer Microspheres with Distance from the Injection Well

**DOI:** 10.1021/acsomega.5c11511

**Published:** 2026-04-13

**Authors:** Jianbing Li, Xingcai Wu, Gang Shi, Liwei Niu

**Affiliations:** † School of Petroleum Engineering, 12556 Hebei Petroleum University of Technology, Chengde, Hebei 067000, People’s Republic of China; ‡ College of Chemistry and Chemical Engineering, 117792 Northeast Petroleum University, Daqing, Heilongjiang 163318, People’s Republic of China; § Startwell (Beijing) Technology Co., Ltd., Beijing 100190, People’s Republic of China

## Abstract

When mobility control agents migrate over long distances in reservoirs, their mobility control ability varies with the distance from the injection well owing to shear degradation, adsorption, and retention. The mobility control ability of a chemical agent is commonly quantified using the resistance factor (RF). To investigate the variation in the RF with migration distance from an injection well, an 18-m-long artificial core was divided into ten segments herein, and the RFs of a polymer solution (partially hydrolyzed polyacrylamide; HPAM) and a polymer microsphere (PM) dispersion system flowing through each segment were independently measured. Experimental results showed that with increasing migration distance, the hydrodynamic radius (*D*_h_) and the RF of HPAM decreased continuously. Conversely, the particle size (*d*) and the RF of PMs initially increased and then decreased. Based on the observed RF variation, a dependency relationship was established between the RF and four parameters: the maximum resistance factor (RF_M_), migration distance (*L*), distance to peak RF (*L*_0_), and decay coefficient (ω). Further analysis revealed that increasing the injection rate from 0.3 to 0.7 mL min^–1^ reduced the RF_M_ of PMs from 29.7 to 24.6, and increased *L*_0_ from 7.2 to 14.4 m and ω from 0.0166 to 0.0271 m^–1^, indicating a faster decline in the RF. Consequently, the projected RF at 100 m from the injection well (RF_100_) decreased from 7.1 to 3.3. When the PM concentration increased from 0.2 to 0.4 wt %, the RF_M_ of the surfactant/polymer microsphere (S/PM) system increased from 16.2 to 41.3, *L*_0_ remained constant at 10.8 m, ω increased from 0.0151 to 0.0179 m^–1^, and the projected RF_100_ increased from 5.0 to 9.2. When the migration distance *L* > 17.1 m, the projected RF of the S/PM system exceeded that of the PM system, with a projected RF_100_ difference of 2.3. These results demonstrated that the addition of surfactant extended the effective range of PMs. The proposed method provides a new approach for optimizing the selection and design of chemical mobility control agents.

## Graphical abstract

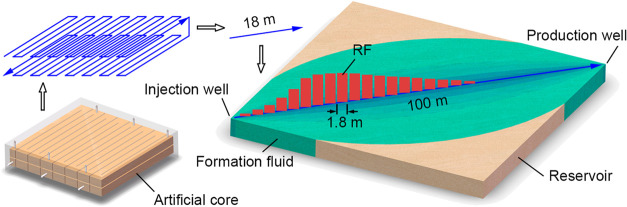

## 1 Introduction

To enhance waterflood sweep efficiency, high-molecular-weight polymers are commonly added to injection water to increase the water-phase viscosity and reduce the mobility ratio, thereby mitigating viscous fingering.^1^ Among these polymers, partially hydrolyzed polyacrylamide (HPAM) is the most widely used mobility control agent in oilfields owing to its abundant availability of raw materials, ease of synthesis, low cost, suitability for large-scale industrial production, and excellent viscosifying performance. HPAM enters high-permeability zones and is adsorbed and retained within pore spaces, reducing water-phase permeability in these layers and adjusting the injection profile, thus improving sweep efficiency.^2^ Compared with conventional water injection, polymer injection increases operational costs, and the magnitude of this increase depends on the polymer concentration.^3,4^ However, polymer flooding yields high additional oil recovery and improvement in the net present value, substantially enhancing the return on investment of oilfield operations.^5,6^ Despite these advantages, HPAM still exhibits several limitations. Under the influence of environmental factors such as high temperature, salinity, and microbial activities, it is highly susceptible to degradation and shrinkage of the molecular chain, causing a loss in viscosity.^7–9^ Furthermore, analyses of produced fluids have revealed that polymer molecules undergo substantial mechanical degradation under shear conditions, limiting their effectiveness in addressing deep-seated water channeling in reservoirs. To overcome these challenges, various deep-profile control agents have been developed, including weak gels,^10,11^ colloidal dispersion gels,^12^ preformed particle gels,^13^ and polymer microspheres (PMs).^14^ Among these technologies, PMs have emerged as rapidly advancing, promising deep-profile control agents with broad application potential. PMs exhibit excellent thermal stability, controllable particle sizes, good swelling capacity, and strong salt resistance.^15–18^ Moreover, PMs can be synthesized through several polymerization techniques, including dispersion, emulsion, and suspension polymerization. Compared with linear polymers, PM dispersion systems exhibit lower viscosity and smaller pre-expansion particle sizes, enabling them to migrate more easily into deep reservoir zones along with injected fluids. Once in place, PMs can block pore throats through mechanisms such as adsorption and deposition, pore-throat plugging, accumulation plugging, and bridging plugging, thereby increasing water flow resistance and diverting injected fluids toward unswept regions.^19,20^ Field applications have demonstrated significant quantitative benefits. For example, pilot tests indicated a 14% uplift in oil production over the first six months, with peak oil rates increasing by approximately 25%.^21^ In another case, the treatment was effective in 11 wells, resulting in a total incremental oil production of 1365.5 tons over an average validity period of 150 days.^22^

Effective suppression of viscous fingering and mitigation of deep-seated water channeling require chemical agents to maintain strong mobility control ability in deep reservoir zones. The resistance factor (RF), defined as the ratio of pressure drop during chemical injection to that during water injection, is commonly used to characterize the flow resistance of chemical agents. The RF is widely used to evaluate the migration and plugging behavior of PMs,^14,23^ and is typically measured through laboratory physical simulation, as summarized in Table 1.

**Table 1:** Summary of Methods to Measure the RF of PMs^a^

injection method	references	*t*_max_ (d)	physical model (dimension, cm)	injection rate (mL min^–1^)	data description
a. Direct injection (No preswelling): The PM dispersion is prepared and immediately injected into the core without preswelling.	Du et al.^24^	10	Ø3.8 × 10	0.5	RF evaluated for different particle sizes, concentrations, permeabilities, and surfactant effects.
	Jin et al.^25^	15	Ø3.8 × 8	0.5	
	Shi et al.^26^	>0.38	Ø2.5 × 5	0.1	
	Li et al.^14^	7	Ø2.5 × 10	0.3	
	Zhao et al.^27^	7		0.5	
b. Injection after preswelling: The PM dispersion is prepared and allowed to swell in a container for a specific period before injection.	Wang et al.^28^	1	Ø2.5 × 20	0.4	RF measured for the same microspheres at different swelling times and particle sizes.
	Jia et al.^22^	13	Ø2.5 × 10	0.3	
	Zhao et al.^29^	13	Ø2.5 × 10	0.3	
	Li et al.^14^	7	Ø2.5 × 10	0.3	
	Yu et al.^30^	2	Ø2.5 × 5	0.5	
c. In situ swelling: The PM dispersion is injected into the core without preswelling, and then the core is kept static to allow *in situ* swelling.	Liu et al.^31^	8	Ø 2.5 × 10	0.9	Pressure response measured after PM swelling within the core.
	Zhu et al.^32^	>0.5	—/30	1.0	

a *t*_max_ represents the time (days) required for PMs to swell to their maximum size.

Currently, the RF of the PM dispersion system is commonly measured using three experimental methods

In the first method, the PM dispersion system is directly injected into the core. This approach allows the RF to be measured under different matching conditions, such as varying initial PM particle sizes, core permeabilities, and the presence or absence of surfactants, thereby enabling assessment of compatibility between PMs and the reservoir. However, the RF obtained using this method corresponds only to the initial state of the PMs, which is largely different from the actual value, because the PMs are swellable.

In the second method, the PM dispersion system is allowed to stand for a certain period before core injection, enabling partial swelling before flooding. This method can be used to determine the RF of PMs after different swelling times. Nevertheless, it cannot simulate the dynamic transport behavior of PMs or the dynamic swelling process during transport under reservoir conditions. In addition, the effect of shear during flow is not adequately considered.

In the third method, the PM dispersion system is injected directly into the core, held under controlled pressure and temperature conditions for a specific period to allow in situ swelling, and then subjected to subsequent water flooding. This approach enables measurement of the flooding pressure of statically swelled PMs within porous media. However, it cannot measure the actual RF during migration and shares the same drawbacks as the second method: it cannot simulate the dynamic transport and swelling behavior of PMs under reservoir conditions, and it neglects shear effects.

The cores used in these methods to study the RF of PMs are relatively short. Therefore, it is reasonable to assume that the particle size of PMs remains unchanged during migration within short cores. However, during long-distance migration in reservoirs, PMs are continuously subjected to shear, adsorption, and retention, leading to progressive changes in particle sizes and concentrations and, subsequently, gradual changes in RFs. In chemical flooding operations, the typical spacing between injection and production wells ranges from 75 to 300 m, indicating that short-core experiments cannot adequately capture the full migration of PMs in reservoirs. Long-core flooding experiments with multiple pressure measurement points provide a more realistic means of simulating performance changes during the migration of chemical agents. For example, long-core studies have been used to investigate the strength of inorganic gels formed by the OMGL system and Ca^2+^ in a porous medium and its influencing factors.^33^ However, studies focusing on the variation of the RF of chemical agents at different migration distances from the injection well remain scarce.

To address this gap, this study investigates RF variation at different migration distances during the implementation of HPAM and PM flooding in oil fields. An 18-m-long artificial core was uniformly divided into ten segments, and the RFs of HPAM and PMs were determined separately as they flowed through each segment. Based on the observed RF variation characteristics, an RF dependency model was established, and the differences between HPAM and PMs were comparatively analyzed. In addition, the effects of injection rate, PM concentration, and surfactant addition on the RF model were evaluated. In this work, a large-scale physical simulation method was used for the first time to reveal the changing trends of RF for HPAM and PMs during reservoir migration. The proposed method offers a more comprehensive and scientifically robust approach to evaluating chemical agent selection, thereby supporting optimized oil recovery strategies and improving decision-making efficiency and accuracy.

## 2 Experimental Section

### 2.1 Materials

The polymer used in this study was HPAM, with a relative molecular weight of 2.5 × 10^7^ and a solid content of 88.1%, supplied by PetroChina Daqing Refining and Chemical Company. The physicochemical properties of the polymer are listed in Table 2. PMs with a solid content of 100% were provided by the Research Institute of Petroleum Exploration & Development. The PMs were synthesized via inverse emulsion polymerization, and their chemical structure is illustrated in Figure 1A.^14^ The betaine surfactant (S) was synthesized in our laboratory, and its chemical structure is shown in Figure 1B.

**Figure 1:**
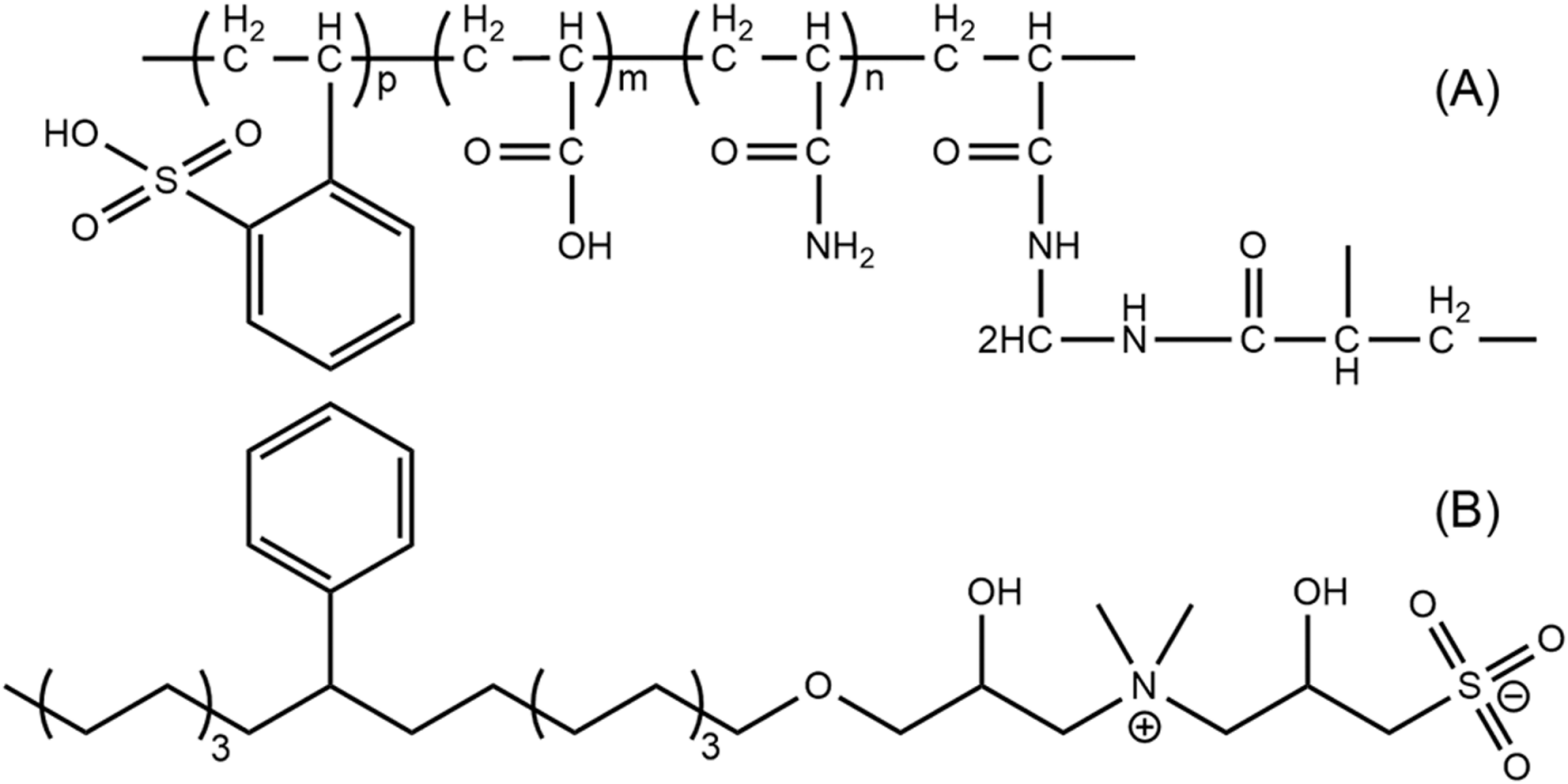
Chemical structures of (A) PMs and (B) betaine surfactant.

**Table 2:** Physicochemical Properties of HPAM

relative molecular weight (Daltons)	solid content (%)	degree of hydrolysis (%)	screen factor	insoluble content (%)	dissolution time (h)
2.5 × 10^7^	88.1	26.5	41.0	0.0992	<2

Formation water and injection water obtained from the Daqing Oilfield were used in all experiments. Before use, both water samples were filtered through 0.22-μm track-etched membranes and fully degassed. Formation water was used to saturate the cores, while injection water was employed for chemical agent preparation and core flooding experiments. The ionic compositions of the water samples are listed in Table 3.

**Table 3:** Ionic Compositions of Formation and Injection Water

	cation (mg L^–1^)			anion (mg L^–1^)				
parameter	Na^+^ + K^+^	Ca^2+^	Mg^2+^	HCO_3_^–^	Cl^–^	SO_4_^2–^	CO_3_^2–^	total salinity (mg L^–1^)
Formation water	2428	14.9	7.48	2160.08	2266.88	54.10	197.66	7156.5
Injection water	1265	32.1	7.30	1708.56	780.12	9.61	210.07	4012.7

A large-scale physical model was constructed using two slotted artificial cores, each measuring 60 cm × 60 cm × 4.5 cm, which were connected in series using epoxy resin casting. The mineral composition of the cores was: quartz (92.18 wt %), orthoclase (3.06 wt %), plagioclase (3.36 wt %), calcite (0.11 wt %), dolomite (0.04 wt %), and clay minerals (1.25 wt %). Fourteen slots were cut into each core to form a serpentine flow path consisting of 15 channels, each 0.6 m in length. The external dimensions of the encapsulated physical model were 63.5 cm 63.5 cm 12.5 cm. This configuration created a continuous flow path of 18 m with a measured water permeability of 0.810 μm^2^ (Figure 2).^14,23^

**Figure 2:**
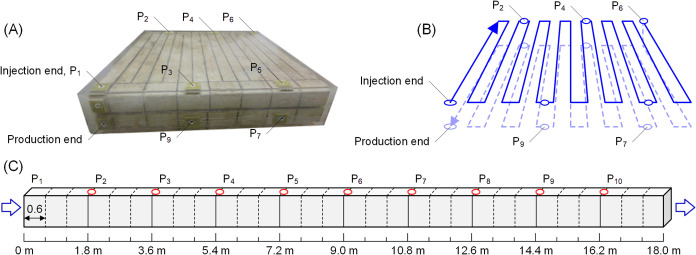
(A) Photograph of the large-scale physical model. (B) Schematic of the serpentine flow path. (C) Equivalent linear flow diagram. Adapted from ref 23 under the terms of the Creative Commons Attribution 4.0 International License (CC BY 4.0).

### 2.2 Preparation of Chemical Agents

#### 2.2.1 Preparation of HPAM Solution

A predetermined volume of injection water was transferred into a beaker and stirred to create a vortex. Dry HPAM powder was gradually added to prepare a 2000 mg L^–1^ polymer solution. The mixture was stirred continuously for 3 h to ensure complete dissolution.

#### 2.2.2 Preparation of PM Dispersion

A specified volume of injection water was placed in a beaker under mechanical stirring. PMs were slowly added to prepare a dispersion with a concentration of 0.3 wt %. The dispersion was stirred for 30 min to obtain a uniform mixture.

#### 2.2.3 Preparation of S/PM Composite System

A calculated mass of injection water was weighed and placed in a beaker under stirring. The surfactant was added to prepare a 0.2 wt % surfactant solution, followed by the addition of PMs to achieve final PM concentrations of 0.2, 0.3, and 0.4 wt %. The mixture was then stirred for 30 min to ensure uniform dispersion.

### 2.3 Chemical Agent Performance Test

The initial particle size of PMs was measured using a Zetasizer Nano ZS dynamic light scattering (DLS) instrument (Malvern, UK), with a measurement range of 0.3 nm–10 μm. Due to the significant increase in particle size after swelling, the particle size of swollen PMs was measured using a Horiba LA-300 laser diffraction particle size analyzer (Horiba, Japan) with a measurement range of 0.1–600 μm. The hydrodynamic radius (*D*_h_) of the polymer molecular coil was determined using a BI-200SM wide-angle dynamic/static light scattering system (Brookhaven Instruments, USA), with a detection range of 10 nm to 1 μm. For all particle size and molecular dimension measurements, samples were diluted with injection water to a suitable concentration to ensure accurate detection. The micromorphology of PMs was examined using a SteREO Discovery.V12 stereomicroscope (Carl Zeiss, Germany) and an S-3400N scanning electron microscope (Hitachi, Japan).

### 2.4 Resistance Factor

The mobility control performance of chemical agents at different migration distances in reservoirs was evaluated by measuring the RF of the chemical agents in each section of the core after shearing.^23,34^ Figure 2 shows that the large-scale physical model is divided into ten sections. After vacuuming and saturating the model with water, the total saturated water volume of the model was measured, while the water permeability of each section was determined using adjacent pressure taps. Assuming that the pore volume is proportional to permeability, the PV of each section was calculated by allocating the total saturated water volume based on the permeability ratio of each section, and this value was then used to determine the porosity. During the experiments, the number of injected PVs was calculated as the ratio of the injected volume to the saturated water volume of the corresponding section. The large-scale physical model, combined with a multifunctional chemical flooding experimental system, was employed. The experimental procedure is illustrated in Figure 3 and consisted of the following steps:

(1) Vacuum the large-scale physical model and saturate it with water. Record the saturated water volume.
(2) Inject water until the pressure stabilizes and record the pressure drop (Δ*P*_w_).
(3) Inject 4–6 PV of the chemical agent and record the stabilized pressure drop (Δ*P*_c_).
(4) Inject 4–6 PV of subsequent water (postflush) and record the pressure drop (Δ*P*_w_).
(5) Calculate the RF and residual resistance factor (RRF) using eqs 1
1 $$\begin{array}{l} \mathrm{RF}=\frac{{\Delta P}_{c}}{{\Delta P}_{w}}\\ \mathrm{RRF}=\frac{{\Delta P}_{\mathrm{sw}}}{{\Delta P}_{w}} \end{array}$$
  where Δ*P*_c_ is the stabilized pressure drop during the chemical agent injection phase (MPa), Δ*P*_w_ is the stabilized pressure drop during the initial water injection phase (MPa), and Δ*P*_sw_ is the stabilized pressure drop during the subsequent water injection phase (MPa).
(6) After pressure stabilization, collect the produced fluids and measure the hydrodynamic radius (*D*_h_) of the polymer and the particle size (*d*) of PMs in the produced fluid from each core section.

**Figure 3:**
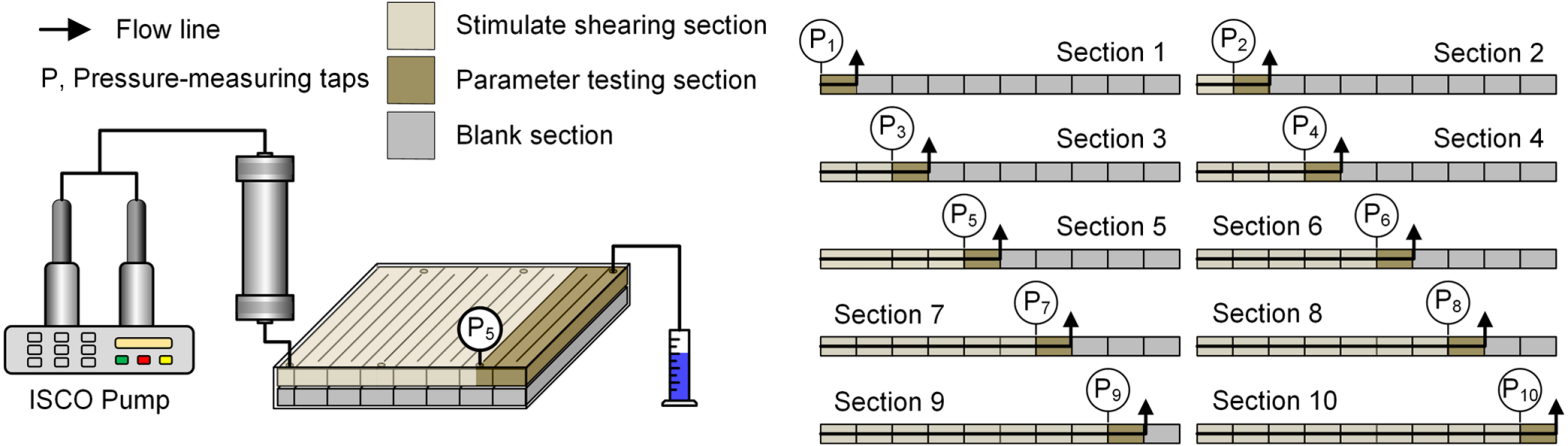
Schematic of the experimental setup for RF measurement.

Polymer solution (*C*_P_ = 2000 mg L^–1^), PM dispersion (*C*_PM_ = 0.3 wt %), and surfactant/PM composite system (*C*_PM_ = 0.3 wt %; *C*_S_ = 0.2 wt %) were used to conduct the experiments. To ensure that PMs maintained a low hydration swelling before entering the core, neither PMs nor S/PMs were preswelled, and fresh samples were reprepared every 2 h during the experiments.

Unless otherwise specified, all experiments were performed at 45 °C with an injection rate of 0.5 mL min^–1^. The polymer solution was presheared to achieve approximately 60% viscosity retention, and PMs and S/PMs were subjected to the same shearing conditions.

## 3 Results and Discussion

### 3.1 Polymer Microsphere Performance

#### 3.1.1 Micromorphology, Particle Size, and Distribution Characteristics

The micromorphology, particle size, and size distribution of PMs in water are shown in Figure 4. As illustrated in Figure 4A,B, the PMs exhibit a regular spherical shape with clear boundaries and are well dispersed in water. The particle size distribution of PMs was mainly concentrated in the range of 0.1–10 μm, with a peak frequency of 21.0%. The median particle size (*D*_50_) was 2.21 μm.

**Figure 4:**
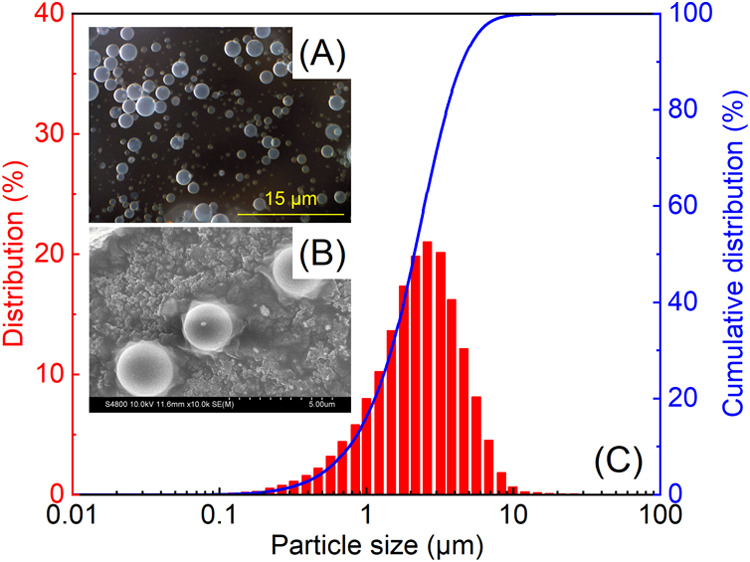
(A) Stereomicroscopy image, (B) scanning electron microscopy (SEM) image, and (C) particle size distribution of PMs.

#### 3.1.2 Water Absorption and Swelling Properties

The relationship between particle size and swelling time for the PMs in the PM system (*C*_PM_ = 0.3 wt %) and the S/PM composite system (*C*_PM_ = 0.3 wt %; *C*_S_ = 0.2 wt %) is presented in Figure 5. The results clearly demonstrate that PMs exhibit pronounced water absorption and swelling behavior, with the particle size of the PMs increasing progressively over time. Rapid swelling occurred immediately after dispersion preparation, followed by a gradual reduction in swelling rate, ultimately reaching equilibrium after approximately 7 days. Notably, at the same swelling time, the particle size of PMs in the presence of surfactant was consistently smaller than that in the PM system. This behavior is attributed to the adsorption of betaine surfactant molecules onto the PM surface through hydrogen bonding and hydrophobic interactions. The adsorbed surfactant reduces the thickness of the hydration layer surrounding the surface carboxyl groups of PMs, hindering hydration and leading to restricted swelling. After 7 days, the median particle size of PMs reached 15.11 μm, corresponding to a 6.84-fold increase relative to the initial particle size. In comparison, the median particle size of the S/PM system reached 14.26 μm, representing a 6.46-fold increase. These results indicate that both PM and S/PM systems possess strong swelling capabilities.

**Figure 5:**
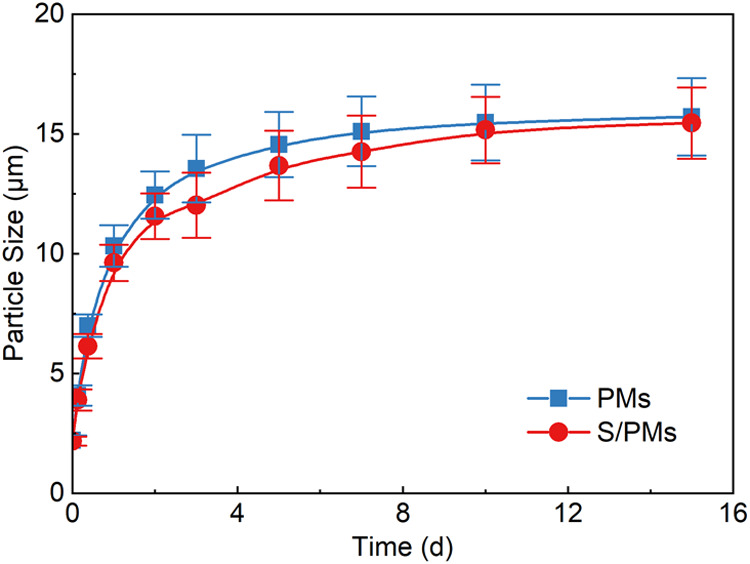
Relationship between particle size and swelling time for PM and S/PM systems.

### 3.2 Resistance Factor

Figure 6A shows the variations of *D*_h_, RF, and RRF of the polymer solution with migration distance. As the migration distance increases, *D*_h_, RF, and RRF all exhibit a decreasing trend. The changes in RF and RRF are closely correlated with the variation in *D*_h_. In aqueous solution, HPAM molecules behave as flexible long-chain macromolecules. When *D*_h_ is relatively large, strong intermolecular entanglement occurs, leaving insufficient time for conformational changes during migration. As a result, polymer molecules are more likely to be trapped in pore throats, leading to higher RF and RRF values. However, as the migration distance increases, polymer chains undergo mechanical degradation (shearing) and adsorption/retention within the formation. Consequently, as *D*_h_ decreases, the degree of physical entanglement weakens, and the likelihood of pore-throat plugging declines, resulting in reduced RF and RRF values.^35^

**Figure 6:**
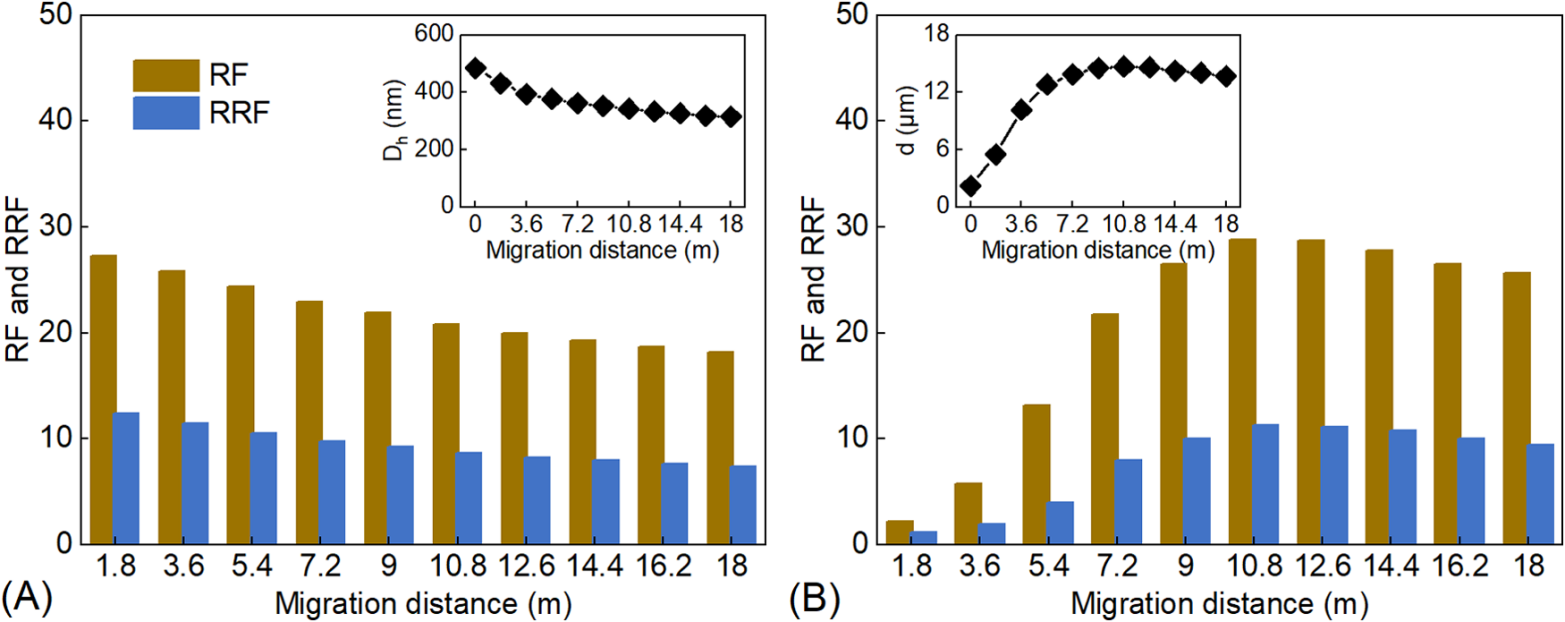
(A) Variations of *D*_h_, RF, and RRF of the HPAM solution with migration distance; (B) variations of particle size (*d*), RF, and RRF of the PM dispersion with migration distance.

Figure 6B shows the relationship between particle size (*d*), RF, and RRF of PMs as a function of migration distance. Unlike the polymer solution, PMs exhibit a characteristic “increase and then decrease” trend in *d*, RF, and RRF with increasing transport distance. In the early sections of the core, PMs exhibit relatively small *d*, making individual particle plugging unlikely and reducing the probabilities of accumulation and bridging plugging. Consequently, the injection pressure remains low, resulting in small RF and RRF values. As PMs migrate deeper, they undergo simultaneous transport and swelling within the core, leading to a continuous increase in *d*. This enhances the probability of accumulation and bridging plugging, thereby increasing the injection pressure and elevating RF and RRF. The values of RF, RRF, and *d* reached their maximums at a migration distance of 10.8 m from the injection end. The value of *d* is determined by the competition between hydration swelling and shear degradation within the core. Previous studies have shown that increasing *d* intensifies shear effects on microspheres.^23^ When the migration distance exceeds 10.8 m, core shear becomes the dominant factor, leading to a decrease in particle size (*d*) and a reduction in RF and RRF. The consistent variation trends of RRF and RF further confirm that the mobility control ability is primarily governed by the physical retention of PMs.

Figure 7 presents the relationships between injection pressure and PV for the polymer and PMs at different sections of the large-scale physical model. During the chemical injection stage, the injection pressure increased with PV and gradually stabilized. During the subsequent water flooding, distinct flow behaviors were observed. In the polymer solution, continued water injection flushed out the polymer, decreasing the injection pressure until it stabilized. In contrast, for PMs within the distance of 10.8 m from the injection end, the injection pressure exhibited a “decrease–increase–stabilize” trend. Beyond 10.8 m, the injection pressure decreased and leveled off. This behavior arises from the competing effects of PM washout and in situ swelling. The washout of PMs reduces flow resistance and injection pressure, whereas the continued swelling of retained PMs in pore throats increases flow resistance. The observed pressure variation reflects the combined influence of these two mechanisms, with washout playing the dominant role, as indicated by the relatively small pressure rebound.

**Figure 7:**
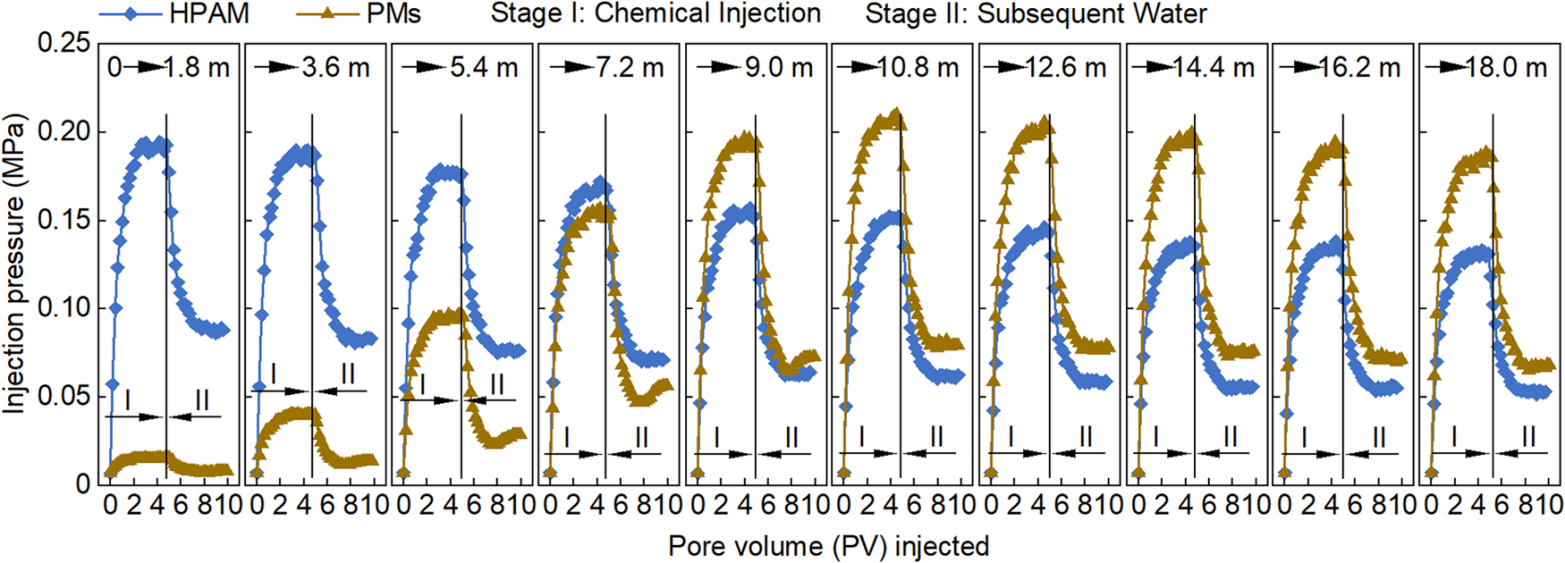
Relationships between injection pressure and PV for the HPAM solution and PMs at different sections of the large-scale physical model.

Overall, within 7.2 m from the injection end, the injection pressure of the polymer was markedly higher than that of PMs. However, the pressure difference between the two systems gradually narrowed as the migration distance extended. Beyond 9.0 m from the injection end, the injection pressure of PMs was higher than that of the polymer, and this difference persisted until 18 m. This behavior is attributed to the substantial swelling of PMs at this distance, which enhances accumulation and bridging plugging, thereby increasing flow resistance. By contrast, the shear-degraded polymer molecules exhibit a lower *D*_h_ value, diminished physical entanglement, and more flexible molecular conformations during migration. This facilitates easier passage through pore spaces, thereby reducing the injection pressure. These results demonstrate that, unlike polymer solutions, PMs are capable of attaining optimal mobility control in the middle and deep sections of the core. This characteristic helps mitigate near-wellbore plugging while enhancing the mobilization of residual oil in deep reservoir zones, which is of great significance for enhanced oil recovery.

### 3.3 Relationship between Resistance Factor and Migration Distance

During the migration of chemical agents in reservoirs, increased migration distance leads to progressive adsorption, retention, and shear degradation, eventually rendering them ineffective. After long-distance migration, the RF gradually decreases and approaches unity. This process can be approximately described using an adjusted exponential function (eq 2)­

2 $$y=a\;e^{-\mathrm{bx}}+1$$

By substituting the physical parameters into eq 2, the relationship between RF and migration distance (*L*) of the chemical agent in porous media is obtained (eq 3)­

3 $$RF=\left({RF}_{M}-1\right)e^{-\omega L}+1$$

where RF_M_ is the maximum resistance factor attained during migration in the reservoir (dimensionless); ω is the decay coefficient (m^–1^), which reflects the rate of decline in RF due to adsorption, retention, and shear degradation when the chemical agent migrates through porous media, and *L* is the migration distance (*m*). The parameters RF_M_ and ω depend on the physicochemical properties of the chemical agent, its concentration, and the shear intensity (or pore structure) of the porous medium.

#### 3.3.1 HPAM

Based on the RF measurements of the polymer solution (Figure 6A), the relationship between RF and migration distance was fitted using eq 3 (Figure 8). The resulting fitting equation is given as follows

4 $$RF=\left(29.3-1\right)e^{-0.0261L}+1,\left(R^{2}=0.9698\right)$$

**Figure 8:**
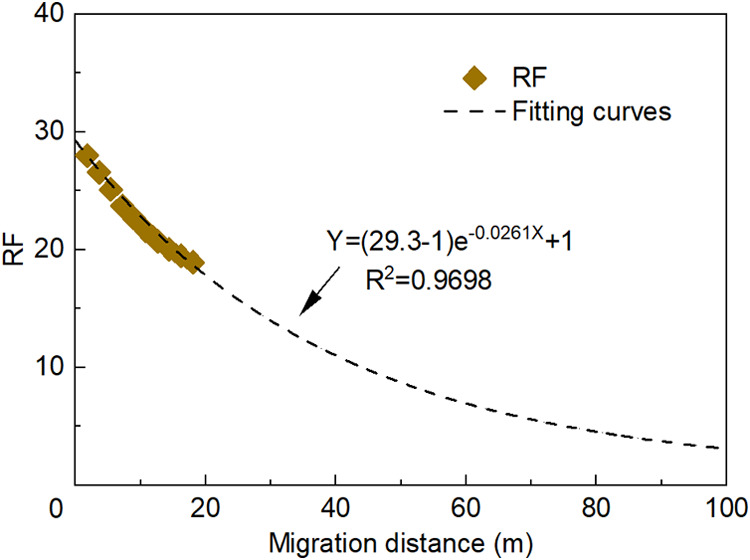
Relationship between RF and migration distance for the polymer solution.

From eq 4, the maximum RF of the polymer is RF_M_ = 29.3, and the decay coefficient is ω = 0.0261 m^–1^. Because the RF of the polymer solution reaches its maximum at the injection point, RF_M_ can also be measured using short-core experiments. Consequently, eq 4 can be employed to estimate the RF of the polymer solution at different distances from the injection well.

#### 3.3.2 PMs

As shown in Figure 6B, the variation of RF for PMs can be divided into two distinct stages: an initial rising stage followed by a declining stage.

During the RF rising stage (Figure 9A), the preliminary curve-fitting result yielded a minimum RF value of less than unity. However, since RF is always ≥ 1 and the initial particle size of PMs is very small (corresponding to an initial RF of ≈1), the intercept was constrained to unity. The corrected fitting equation for the rising stage is expressed as eq 5

5 $$RF=-0.0587L^{3}+0.9989L^{2}-1.3635L+1,\left(R^{2}=0.9981\right)$$

**Figure 9:**
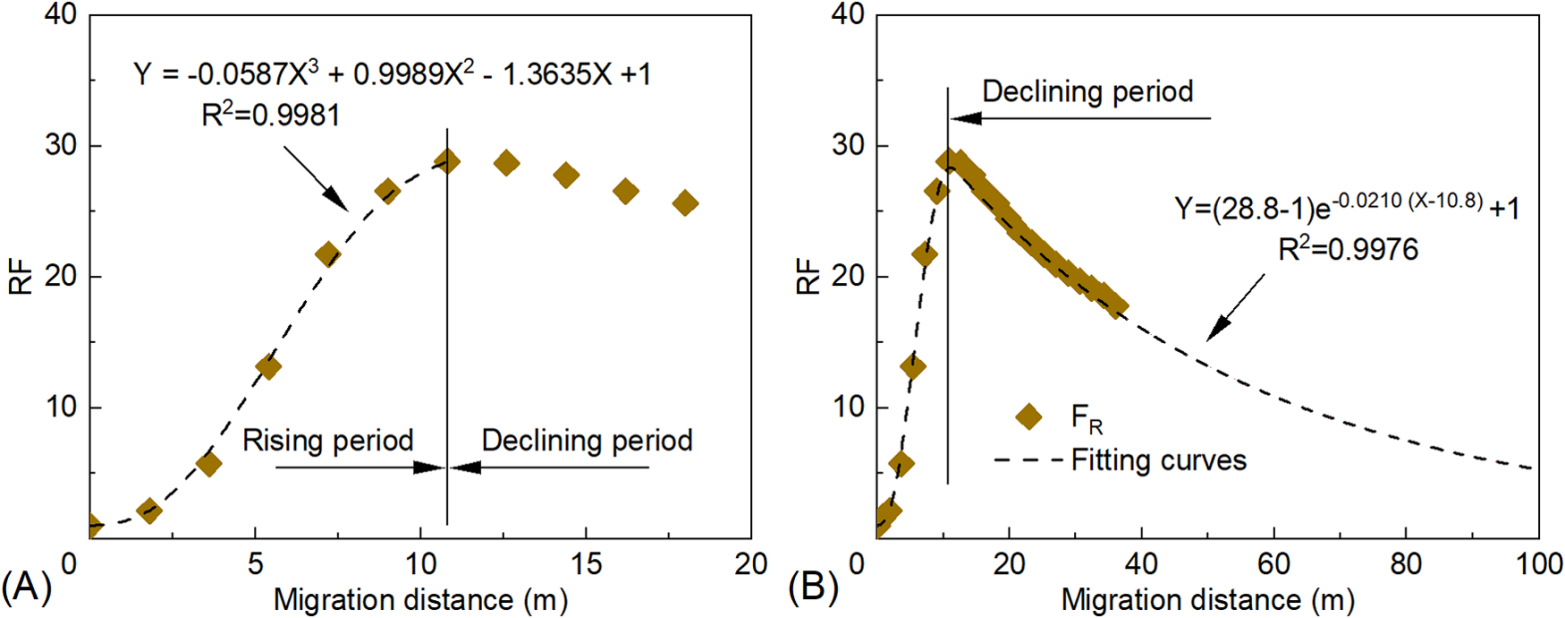
Relationships between RF and migration distance for PMs: (A) RF rising stage and (B) RF declining stage.

In the RF declining stage, the relationship between RF and migration distance of PMs is shown in Figure 9B. Curve fitting was performed according to eq 3, yielding eq 6

6 $$RF=\left(28.8-1\right)e^{-0.021\left(L-10.8\right)}+1,\left(R^{2}=0.9976\right)$$

Under the given experimental conditions, the value 10.8 in eq 6 is a constant. Hence, eq 6 can be generalized as eq 7

7 $$RF=\left({RF}_{M}-1\right)e^{-\omega \left(L-L_{0}\right)}+1$$

where *L*_0_ is the distance corresponding to maximum RF (m). Unlike HPAM, the RF_M_ of PMs occurred in the middle of the core. This behavior is primarily governed by the maximum particle size (*d*) of PMs after swelling and the shear intensity of the core. When is relatively small, the shear intensity of the core is also lower. Therefore, if the shear effect of the core during the swelling process of PMs is neglected, short-core experiments can be used to measure the RF of fully swollen PMs, which can then be used as a reasonable approximation of RF_M_.

According to eq 7, the RF_M_ of PMs is 28.8, the decay coefficient is ω = 0.021 m^–1^, and the distance to peak RF is *L*_0_ = 10.8 m. Specifically, *L*_0_ reflects the impact of water absorption and swelling properties of the PM on RF, representing the migration distance of PMs in the porous medium during the RF rising stage (water absorption–swelling period). Thus, *L*_0_ reflects the swelling speed of PMs in porous media. During the migration of PMs, *L*_0_ can be estimated using eq 8

8 $$L_{0}\approx \frac{Qt_{\max}}{A}$$

where *Q* is the injection rate (m^3^ d^–1^), *t*_max_ is the time required for PMs to swell to their maximum particle size (days), and *A* is the cross-sectional area of the flow channel (m^2^).

Equation 8 indicates that *L*_0_ is directly proportional to both *Q* and *t*_max_. Hence, the deep migration capability of PMs can be further enhanced by prolonging the swelling time through chemical synthesis or by extending the distance to peak RF (*L*_0_) through higher injection rates.

#### 3.3.3 Comparison of HPAM and PMs

According to Sections 3.3.1 and 3.3.2, the relationships between RF and migration distance for the polymer solution and PMs can be described by eq 9 and illustrated in Figure 10.

9 $$RF=\{\begin{array}{l} f\left(L\right)=aL^{3}+bL^{2}+\mathrm{cL}+1,,L<L_{0}\\ g\left(L\right)=\left({RF}_{M}-1\right)e^{-\omega \left(L-L_{0}\right)}+1,,L\geq L_{0} \end{array}$$

**Figure 10:**
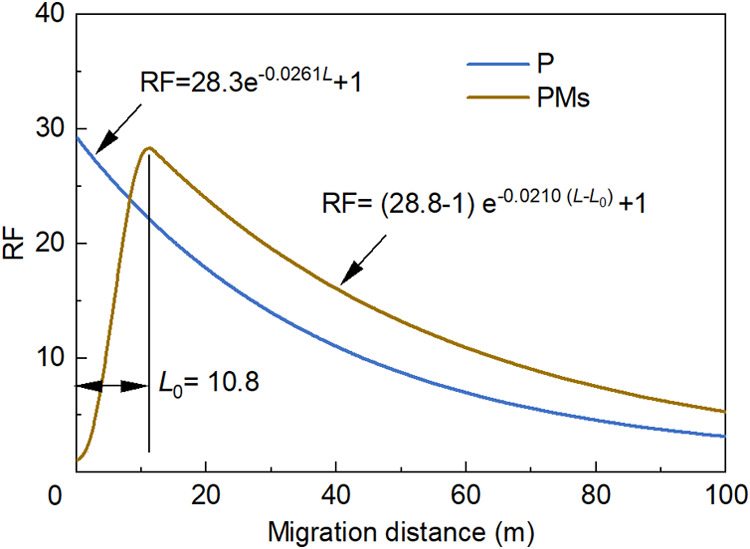
Relationships between RF and migration distance for the polymer solution and PMs.

For the polymer solution, *L*_0_ = 0; thus, its RF follows the function g (*L*). By contrast, for PMs, *L*_0_ is a constant under certain conditions. Thus, RF follows *f*(*L*) when *L* < *L*_0_, RF follows *g*(*L*) when *L* ≥ *L*_0_, and RF ≈ RF_M_ when *L* = *L*_0_. Generally, the RF of a chemical agent at different distances from the injection well is jointly governed by three parameters: RF_M_, *L*_0_, and ω.

Under the experimental conditions employed, HPAM and PMs exhibited similar RF_M_ values, as shown in Figure 10. However, compared with the polymer solution, the RF curve of PMs is shifted rightward by 10.8 m, corresponding to the value of *L*_0_. Furthermore, PMs exhibit a smaller ω value than HPAM, indicating a slower decline in RF with migration distance. Under the combined effects of the above factors, when *L* > *L*_0_, the RF curve of the PMs is positioned entirely above that of the polymer solution, demonstrating that PMs possess stronger deep profile control ability than HPAM.

### 3.4 Resistance Factor of PMs at Different Migration Distances

#### 3.4.1 Effect of Injection Rate on the Resistance Factor

Figure 11 illustrates the relationships between injection pressure and PV during the RF tests of PMs conducted at different injection rates. At a given injection rate, the injection pressure exhibited a characteristic trend: initially increasing, then decreasing with increasing migration distance. This behavior is attributed to the combined effects of hydration swelling and shear degradation. As PMs migrate and absorb water, their particle size (*d*) increases, leading to higher flow resistance and injection pressure. When PMs reach their maximum swollen size, the injection pressure also reaches a maximum value (*P*_max_). Beyond this point, shear effects within the core dominate, reducing particle size (*d*) and leading to a corresponding decline in injection pressure.

**Figure 11:**
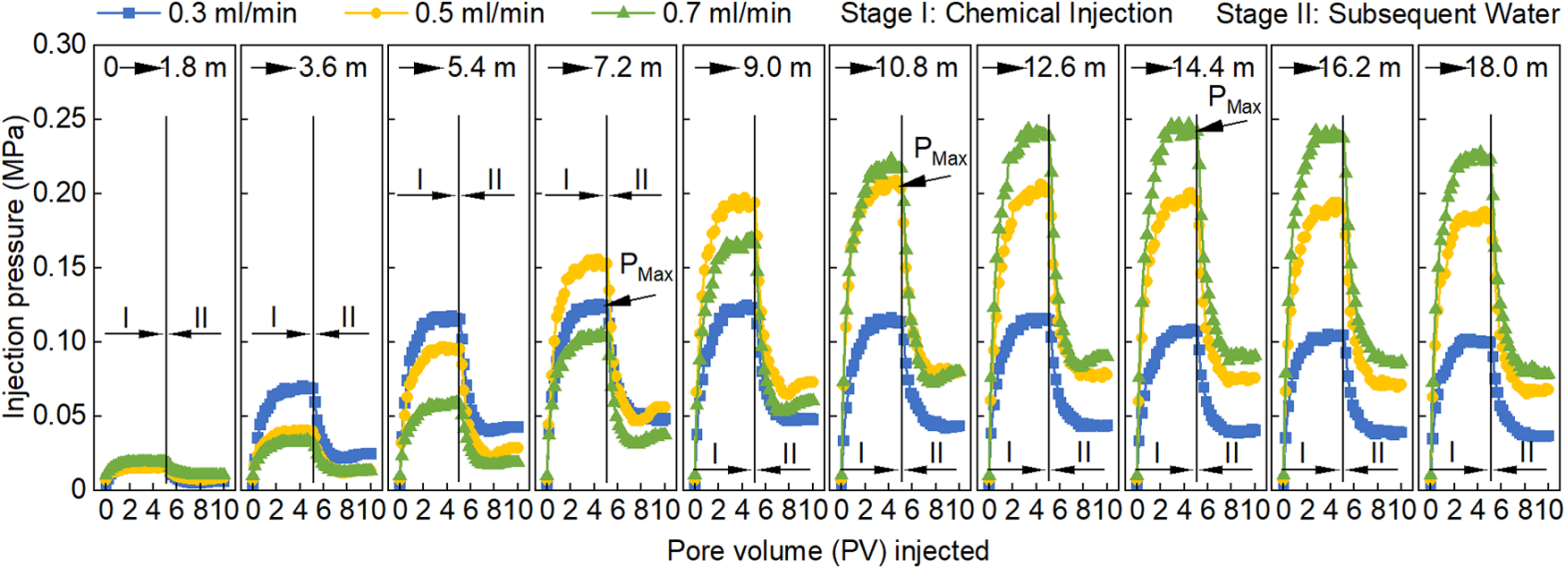
Relationships between injection pressure of PMs and PV at different injection rates.

Generally, a higher injection rate results in a longer migration distance corresponding to *P*_max_. Specifically, at injection rates of 0.3, 0.5, and 0.7 mL min^–1^, the maximum injection pressure occurred at migration distances of 7.2, 10.8, and 14.4 m from the injection end, respectively. This behavior arises because PMs behave as elastic spheres: a higher injection rate imposes a larger pressure gradient, enabling PMs to penetrate deeper into the core and shifting the location of maximum resistance farther from the injection end.

Moreover, Figure 11 shows that within 5.4 m from the injection end, higher injection rates correspond to lower injection pressures, whereas beyond 10.8 m, higher injection rates result in higher injection pressures. Particle size is a key factor controlling injection pressure. Within the near-injection region (5.4 m), PMs are still swelling. At higher injection rates, PMs experience shorter swelling time at the same migration distance, resulting in smaller particle size, lower RF values, and lower injection pressure. Beyond 10.8 m, PMs at all injection rates have completed their swelling process, and injection pressure increases with injection rate due to enhanced flow resistance (following Darcy’s law).

The relationship between RF and migration distance for PMs at different injection rates is shown in Figure 12. During the RF rising stage, higher injection rates lead to a slower increase in RF and lower maximum RF values. For injection rates of 0.3, 0.5, and 0.7 mL min^–1^, the fitted relationships during the RF rising stage are given by eqs 10–12, respectively

10 $$RF=-0.2453L^{3}+2.5879L^{2}-1.908L+1,\left(R^{2}=0.9996\right)$$

11 $$\mathrm{RF}=-0.0587L^{3}+0.9989L^{2}-1.3635L+1,\left(R^{2}=0.9981\right)$$

12 $$\mathrm{RF}=-0.0207L^{3}+0.4796L^{2}-0.9424L+1,\left(R^{2}=0.9954\right)$$

**Figure 12:**
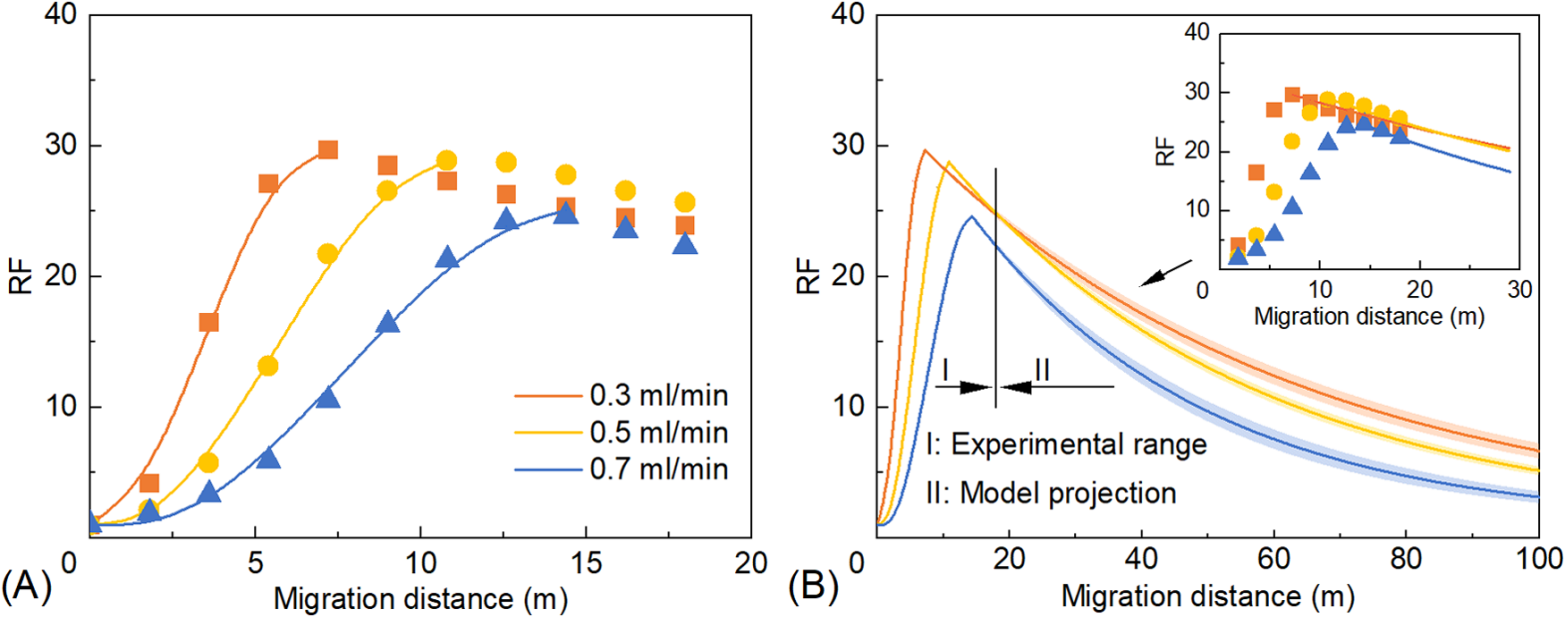
Relationships between RF and migration distance of PMs at different injection rates: (A) RF rising stage and (B) RF declining stage. Shaded areas represent 95% confidence bands. Note that values beyond 18 m (e.g., RF_100_) are model-based projections rather than direct experimental measurements.

During the RF declining stage, an extended migration distance leads to a more rapid decrease in RF at higher injection rates, accompanied by lower maximum RF values (Figure 12B). The fitted relationships for injection rates of 0.3, 0.5, and 0.7 mL min^–1^ are shown in eqs 13–15, respectively

13 $$\mathrm{RF}=\left(29.7-1\right)e^{-0.0166\left(L-7.2\right)}+1,\left(R^{2}=0.9682\right)$$

14 $$\mathrm{RF}=\left(28.8-1\right)e^{-0.0210\left(L-10.8\right)}+1,\left(R^{2}=0.9976\right)$$

15 $$\mathrm{RF}=\left(24.6-1\right)e^{-0.0271\left(L-14.4\right)}+1,\left(R^{2}=0.9593\right)$$

According to eqs 13–15, increasing the injection rate leads to a reduction in the RF_M_ of PMs, accompanied by an increase in the distance to peak RF (*L*_0_) and the decay coefficient (ω). At injection rates of 0.3, 0.5, and 0.7 mL min^–1^, the corresponding values of RF_M_ were 29.7, 28.8, and 24.6, *L*_0_ was 7.2, 10.8, and 14.4, and ω was 0.0166, 0.0210, and 0.0271 m^–1^, respectively. According to eqs 8, increasing the injection rate *Q* directly increases *L*_0_. This indicates that as the PMs reach the position of maximum RF, their RF_M_ decreases due to cumulative shear effects over a longer migration distance. In addition, higher injection rates correspond to a higher pressure gradient and stronger shear intensity, which further contribute to the increase in ω. Based on the pressure analysis during Stage II (Figure 11), increasing the injection rate from 0.3 mL min^–1^ to 0.7 mL min^–1^ reduced the RRF at the outlet (18 m) from 8.8 to 7.8. This decline further confirms that the physical retention ability of PMs is weakened under high-velocity flushing, consistent with the increase in the decay coefficient (ω).

Generally, low injection rates result in high RF values and strong deep-plugging ability of PMs. However, as the experimental data were collected within a migration distance of 0–18 m, RF values at longer distances were obtained through model-based extrapolation using the fitted declining-stage equations. The projected resistance factor at 100 m from the injection well (RF_100_) decreased from 7.1 to 3.3 as the injection rate was increased from 0.3 to 0.7 mL min^–1^. However, at a lower injection rate, the oil production rate is slower and the project duration is longer, which will significantly increase operating costs. Therefore, the PM injection rate should be optimized based on specific reservoir conditions.

#### 3.4.2 Effect of Surfactant on the Resistance Factor

Figure 13 illustrates the relationship between injection pressure and PV for PM and S/PM systems during RF tests at an injection rate of 0.5 mL min^–1^. Compared with the S/PM system, the PM system exhibits higher injection pressure. However, the pressure difference between the two systems gradually decreases with increasing migration distance. The behavior is primarily governed by variations in PM particle size (*d*), which are controlled by the combined effects of hydration swelling and core shearing. Before PMs reach their maximum swollen particle size, swelling dominates particle size evolution. At the same swelling time, PMs in the S/PM composite system exhibit smaller particle sizes than those in the PM system, resulting in lower injection pressures. Once PMs reach their maximum particle size, shear degradation becomes the main factor influencing particle size. Since the presence of surfactant enhances the shear resistance of the microspheres, the difference in injection pressure between the PM and S/PM systems gradually diminishes with increasing migration distance.

**Figure 13:**
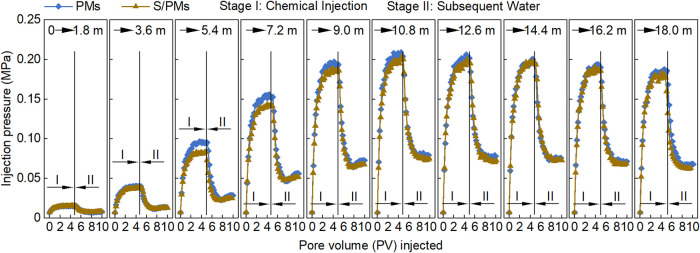
Relationship between injection pressure and PV for PM and S/PM systems.

Figure 14 shows the variation of RF with migration distance for PM and S/PM systems. During the RF rising stage, PMs exhibit a slightly faster increase in RF and a higher RF_M_ compared with the S/PM system, although the overall trends of the two curves are similar (Figure 14A). The relationships between RF and migration distance during the rising stage for the PM and S/PM systems were fitted using eqs 16 and 17, respectively

16 $$\mathrm{RF}=-0.0587L^{3}+0.9989L^{2}-1.3635L+1,\left(R^{2}=0.9981\right)$$

17 $$\mathrm{RF}=-0.0533L^{3}+0.9329L^{2}-1.3588L+1,\left(R^{2}=0.9976\right)$$

**Figure 14:**
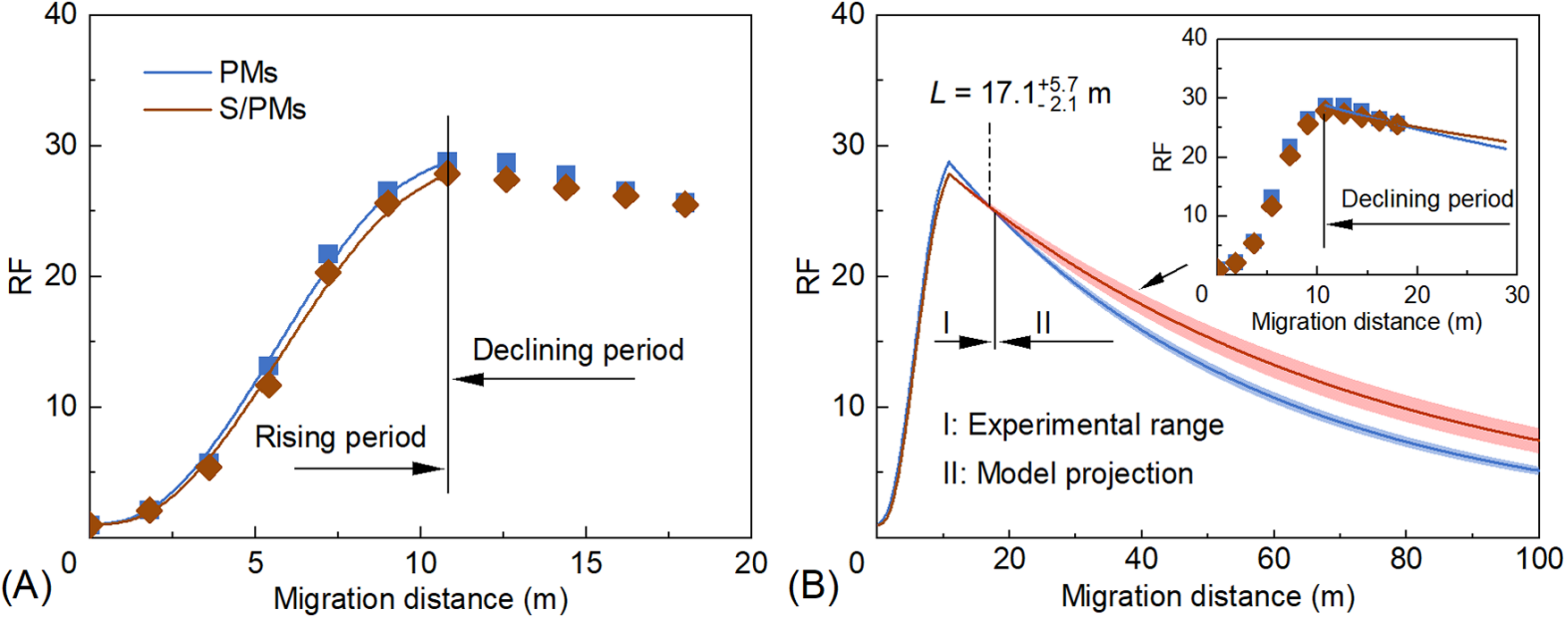
Relationships between RF and migration distance of PM and S/PM systems: (A) RF rising stage and (B) RF declining stage. Shaded regions represent 95% confidence bands. Note that the projected crossover point and values beyond 18 m (e.g., RF_100_) are model-based projections rather than direct experimental measurements.

During the RF declining stage (Figure 14B), the relationships between RF and the migration distance of PM and S/PM systems were described by eqs 18 and 19, respectively

18 $$\mathrm{RF}=\left(28.8-1\right)e^{-0.0210\left(L-10.8\right)}+1,\left(R^{2}=0.9976\right)$$

19 $$\mathrm{RF}=\left(27.9-1\right)e^{-0.0158\left(L-10.8\right)}+1,\left(R^{2}=0.9851\right)$$

According to eqs 18 and 19, compared to the PM system, the S/PM system exhibits a lower RF_M_ and a smaller decay coefficient ω, while the distance to peak RF (*L*_0_) remains unchanged. Although the addition of surfactant reduces the maximum particle size of the microspheres in the S/PM system, resulting in a slightly lower RF_M_ than that of the PM system, it substantially enhances the shear resistance of the microspheres. As a result, the S/PM composite system displays a smaller ω than the PM system.^36^ To further validate this improvement in shear stability, the variation in RRF was evaluated using the Stage II pressure data shown in Figure 13. As the fluid migrated from the position of maximum resistance (10.8 m) to the outlet (18 m), the RRF of the PM system decreased from 11.3 to 9.4, representing a retention loss of 16.8%. In contrast, in the S/PM system, the RRF declined from 10.4 to 9.1, corresponding to a retention loss of 12.5%. This comparison provides additional evidence that the S/PM system maintains better stability and shear resistance during migration.

Consistent with the projection methodology described in Section 3.4.1, a theoretical crossover point is predicted at a migration distance of approximately 17.1 m based on the 95% confidence bands (Figure 14B). Beyond this distance, the S/PM system is projected to maintain a higher RF than the PM system. The projected RF_100_ for the S/PM and PM systems is 7.6 and 5.3, respectively, yielding a difference of 2.3. Despite the inherent uncertainty of long-distance extrapolation, these results indicate that adding surfactant effectively extends the functional range of PMs in deep reservoir zones.

#### 3.4.3 Effects of PM Concentration on the Resistance Factor

Figure 15 shows the relationship between injection pressure and PV during RF tests of the S/PM system at different PM concentrations. As the migration distance increases, the injection pressure exhibits a characteristic trend of “initially increasing and then declining.” Higher PM concentrations increase the likelihood of accumulation and bridging plugging within the core, leading to higher injection pressures.

**Figure 15:**
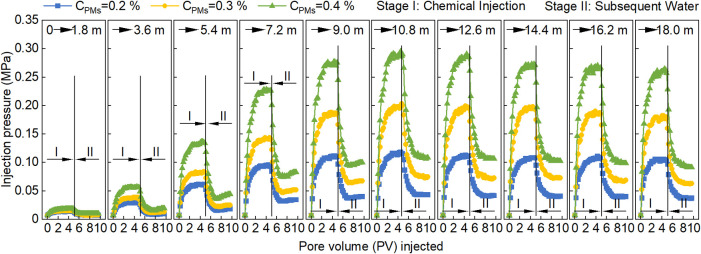
Relationship between injection pressure and PV for the S/PM system at different PM concentrations.

Figure 16 shows the variation of RF with migration distance for the S/PM system at different PM concentrations. During the RF rising stage, higher PM concentrations lead to a more rapid increase in RF and a larger RF_M_ (Figure 16A). The relationships between RF and migration distance of the S/PM system at PM concentrations of 0.2, 0.3, and 0.4 wt % were fitted using eqs 20–22, respectively

20 $$\mathrm{RF}=-0.0341L^{3}+0.5564L^{2}-0.6216L+1,\left(R^{2}=0.9983\right)$$

21 $$\mathrm{RF}=-0.0533L^{3}+0.9329L^{2}-1.3588L+1,\left(R^{2}=0.9976\right)$$

22 $$\mathrm{RF}=-0.0908L^{3}+1.5362L^{2}-2.2506L+1,\left(R^{2}=0.9986\right)$$

**Figure 16:**
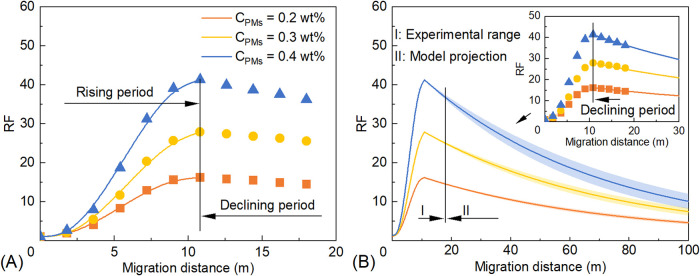
Relationships between RF and migration distance of the S/PM system: (A) RF rising stage and (B) RF declining stage. Shaded regions represent 95% confidence bands. Note that values beyond 18 m (e.g., RF_100_) are model-based projections rather than direct experimental measurements.

During the RF declining stage (Figure 16B), the relationships between RF and migration distance of the S/PM system at PM concentrations of 0.2, 0.3, and 0.4 wt % were fitted using eqs 23–25, respectively

23 $$\mathrm{RF}=\left(16.2-1\right)e^{-0.0151\left(L-10.8\right)}+1\,,\left(R^{2}=0.9922\right)$$

24 $$\mathrm{RF}=\left(27.9-1\right)e^{-0.0158\left(L-10.8\right)}+1,\left(R^{2}=0.9851\right)$$

25 $$\mathrm{RF}=\left(41.3-1\right)e^{-0.0179\left(L-10.8\right)}+1,\left(R^{2}=0.9736\right)$$

According to eqs 23–25, increasing the PM concentration results in higher RF_M_ and ω values for the S/PM system, while *L*_0_ remains unchanged. Specifically, at PM concentrations of 0.2, 0.3, and 0.4 wt %, the corresponding RF_M_ values are 16.2, 27.9, and 41.3, and the ω values are 0.0151, 0.0158, and 0.0179 m^–1^, respectively, with *L*_0_ = 10.8 m in all cases.

Generally, as PM concentration increases, the RF near the injection well markedly increases (Figure 16). When the PM concentration increases from 0.2 to 0.4 wt %, the RF_M_ increases by a factor of 2.55 (from 16.2 to 41.3). According to model projections consistent with the methodology described in Section 3.4.1, the RF_100_ increases by a factor of 1.84 (from 5.0 to 9.2). This behavior can be attributed to two competing effects: increasing PM concentration increases RF_M_, causing an overall upward shift in the RF curve, while simultaneously increasing ω, causing a faster decline in the RF along the migration path.

To elucidate the mechanism behind the increased ω, retention losses were analyzed using the Stage II pressure data (Figure 15). As the fluid migrates from the position of maximum resistance (10.8 m) to the outlet (18 m), the RRF for the 0.2 wt % S/PM system decreases from 6.1 to 5.2, representing a retention loss of 14.8%. In contrast, the RRF of the 0.4 wt % S/PM system reduces from 15.5 to 12.9, yielding a higher retention loss of 16.8%. This increased retention loss indicates that high concentrations experience significantly more intense shearing and washout owing to the high injection pressure.

Consequently, the RF of the S/PM system at different positions is governed by a trade-off between enhanced resistance from increased RF_M_ and accelerated decay from high ω. Although RF_100_ values represent theoretical projections beyond the experimental range, the model predictions with 95% confidence bands further demonstrate that increasing PM concentration is an effective strategy for enhancing mobility control in deep reservoir zones. For clarity, all the fitting equations derived in this study and the corresponding physical parameters are summarized in Table 4.

**Table 4:** Summary of Equations and Parameters

No.	equation	parameter
1	$$\begin{array}{l} \mathrm{RF}=\frac{\Delta P_{c}}{\Delta P_{w}}\\ \mathrm{RRF}=\frac{\Delta P_{sw}}{\Delta P_{w}} \end{array}$$	RF—resistance factor (dimensionless)
		RRF—residual resistance factor (dimensionless)
		Δ*P*_c_—stabilized pressure drop during chemical injection (MPa)
		Δ*P*_w_—stabilized pressure drop during water injection (MPa).
		Δ*P*_sw_—stabilized pressure drop during subsequent water injection (MPa).
2–4	$\mathrm{RF}=\left({\mathrm{RF}}_{M}-1\right)e^{-\omega L}+1$	RF_M_—maximum resistance factor (dimensionless).
		ω—decay coefficient (m^–1^)
		*L*—migration distance (m).
5, 9, 10–12, 16–17, 20–22	$\mathrm{RF}=aL^{3}+bL^{2}+\mathrm{cL}+1\left(L<L_{0}\right)$	*a*, *b*, *c*—fitting coefficients (m ^–3^, m^–2^, and m^–1^, respectively)
		*L*_0_—distance to peak RF (m).
6, 7, 9, 13–15, 18, 19, 23–25	$\mathrm{RF}=\left({\mathrm{RF}}_{M}-1\right)e^{-\omega \left(L-L_{0}\right)}+1\left(L\geq L_{0}\right)$	RF_M_—maximum resistance factor (dimensionless).
		ω—decay coefficient (m^–1^)
		*L*_0_—distance to peak RF (m).
8	$L_{0}\approx \frac{Qt_{\max}}{A}$	*Q*—injection rate (m^3^ d^–1^).
		*t*_max_—time required for PMs to swell to the maximum swollen size.
		*A*—flow cross-sectional area (m^2^).

## 4 Conclusions

When polymer solutions migrate in porous media, increasing migration distance leads to a continuous decrease in hydrodynamic radius (*D*_h_) and RF. In contrast, for PMs, the particle size (*d*) and RF exhibit an “increase-then-decrease” trend.
Based on the RF characteristics, mathematical relationships between RF and migration distance (*L*) were established: RF = *aL* ^3^ + b*L* ^2^ + c*L* + 1 for *L* < *L*_0_, and RF = (RF_M_ – 1)­e^–ω(*L* – *L*0)^ + 1 for *L* ≥ *L*_0_, where RF_M_ is the maximum RF of the chemical agent migrating in the reservoir, *L*_0_ is the distance to peak RF (corresponding to the maximum swollen size), and ω is the decay coefficient (higher ω values correspond to faster RF decay).
Increasing the injection rate reduced the RF_M_ of PMs, increased the distance to peak RF (*L*_0_), and elevated the decay coefficient ω, resulting in faster RF decay. When the injection rate was increased from 0.3 to 0.7 mL min^–1^, RF_M_ decreased from 29.7 to 24.6, *L*_0_ increased from 7.2 to 14.4 m, ω increased from 0.0166 to 0.0271 m^–1^, and the projected RF_100_ reduced from 7.1 to 3.3.
Increasing the PM concentration enhanced the RF_M_ of the S/PM system while leaving *L*_0_ unchanged. When PM concentration increased from 0.2 to 0.4 wt %, RF_M_ rose from 16.2 to 41.3, and the projected RF_100_ increased from 5.0 to 9.2. Furthermore, the addition of surfactant effectively extended the action range of PMs. When the migration distance exceeded 17.1 m, the projected RF of the S/PM system surpassed that of the PM system, achieving an increase of 2.3 at RF_100_, which confirms the superior deep profile control ability of the S/PM composite system.
